# The Effect of Irrigation and Fertilization Reduction on Yield, Quality, and Resource Use Efficiency of Drip-Fertilized Sugar Beet (*Beta vulgaris* L.) in Northern China

**DOI:** 10.3390/plants14040536

**Published:** 2025-02-10

**Authors:** Bowen Zhang, Yuxin Chang, Guolong Li, Shaoying Zhang, Peng Zhang, Zhenzhen Wang, Dejuan Kong

**Affiliations:** 1 College of Agronomy, Inner Mongolia Agricultural University, Hohhot 010019, China; zhangbowen1122@163.com (B.Z.);; 2 Inner Mongolia Ulanqab Institute of Agricultural and Forestry Sciences, Ulanqab 012000, China

**Keywords:** drip fertilization, sugar beet, water and fertilizer reduction, yield, water and fertilizer use efficiency, interaction effect

## Abstract

Sugar beet (*Beta vulgaris* L.) is a crucial economic crop in northern China. Fertilizer and water waste and environmental contaminations were prevalent during sugar beet cultivation. To enhance sugar beet use efficiency and reduce the amount of water and fertilizer required in cultivation, a three-year experiment was performed. Drip fertilization was employed for irrigation and fertilization. The influence of irrigation reductions (15% and 30%) and fertilization reductions (20% and 10%) on yield, sugar content, and water and fertilizer use efficiency was evaluated. The yield of root and sugar was not significantly impacted by the 10% fertilizer and 15% reduction in irrigation relative to the conventional water and fertilizer supply treatment (CK). The yield was significantly reduced by 20% fertilizer and 30% irrigation reduction. The sugar yield was also reduced by 30% through irrigation reduction treatments. The root sugar content was increased by irrigation and fertilization reduction (except 10% fertilizer reduction treatment). Partial factor productivity (PFP) was significantly increased by fertilization reduction. The irrigation water use efficiency (WUEi) was significantly increased by irrigation reduction. Water use efficiency (WUE) and fertilizer agronomic efficiency (FAE) were increased by a 10% reduction of fertilization treatment relative to CK; the impact on FAE was more significant compared to WUE. The FAE was reduced by an irrigation reduction. The WUE was reduced by 20% through fertilization reduction treatments. The influence of each treatment on PFP, FAE, and WUEi of sugar yield was mirrored in yield, but the range was narrowed. To ensure high production and efficiency while conserving water and fertilizer during sugar beet cultivation within the test area, the optimal irrigation amount was 1041.91 to 1286.27 m^3^·ha^−1^and fertilization amount was 394.04 to 408.03 kg·ha^−1^. This investigation offers irrigation and fertilization parameters for drip fertilization of sugar beet and a theoretical foundation for the crop’s water and fertilizer use.

## 1. Introduction

China’s production amounted to 4.11% and 3.42% of the world’s total sugar beet (*Beta vulgaris* L.) production and planting area, respectively [1]. China’s annual sugar beet production was 8.93 million tons, and the planting area was 176.4 thousand ha in 2022 [1]. However, the yield of sugar beet in China was 50.64 t·ha^−1^. This represents a value of 28.83% to 52.32% below the top 10 countries [1]. The growth potential of sugar beet yield is massive in China. Sugar beet is an important source of sugar [2]. The amount of sugar imported to China was 2.29 to 5.66 Mt from 2014 to 2023. The production of sugar could not fully meet demand. The land demand for food and economic crops has limited the increase in sugar beet cultivation. From 2018 to 2022, the sugar beet planting area in China decreased by 9100 ha to 84,100 ha compared to the highest year (2007). Enhancing sugar beet unit production is an important method of increasing sugar supply. Inner Mongolia is a major sugar beet planting area in China; inner Mongolia accounts for 53.61% and 43.11% of the national total sugar beet planting area and production, respectively (China National Bureau of Statistics). However, fertilizer and water overuse, poor soil, and water limitations are pronounced in this region. An important assurance for a high crop yield is an appropriate water and fertilizer supply [3,4,5,6]. Sugar beet productivity and quality are negatively impacted by over-irrigation and fertilization [7,8,9]. It is essential to reduce irrigation and fertilization and improve efficiency during the cultivation of sugar beet.

Studies have suggested that full irrigation and fertilization can produce higher yields, while water and fertilizer use efficiency is reduced [10,11,12,13,14]. Appropriate reduction in fertilizer application and irrigation has no significant impact on crop yield or quality [15,16,17]. In arid climates, Li et al. conducted research suggesting that the sugar content and WUEi of sugar beet root tubers increased alongside decreasing levels of irrigation [18]. Khan et al.’s [19] study revealed no significant difference in yield between treatments with 150 kg·ha^−1^ potassium fertilizer (K_2_O) and 225 kg·ha^−1^. Additionally, Afshar et al. [20] indicated that increasing nitrogen fertilizer application (56–224 kg·ha^−1^) could improve sugar beet yield; however, it significantly reduced sucrose content and had no significant effect on sugar yield.

Numerous studies have indicated that fertilization and irrigation profoundly interact. Nitrogen application could elevate water use efficiency by regulating the anatomical structure and function of plant leaves. Cai et al. [21] and Li et al. [22] applied increasing nitrogen fertilizer to *Arabidopsis* and *Schima*, respectively, under drought stress. The findings demonstrated that the instantaneous water use efficiency of plant leaves was significantly increased by applying nitrogen. Similarly, Yan et al. [23] unveiled that in desert climates, sugar beet yield, sugar yield, and water use efficiency at each irrigation level increased alongside nitrogen application. Ati et al. [24] determined that potato tuber yield and water use efficiency could be effectively enhanced by potassium application under trench irrigation and drip irrigation mode.

Regression analysis was employed to develop and apply the interaction of fertilizer and water. Hu et al. [25] determined that the optimal irrigation and fertilizer quantities for cucumbers grown in substrate bags were 13.54–23.78 g/plant and 37.71–52.59 L/plant, respectively, using multiple regression analysis. Zhang et al.’s [26] study showed the optimal total water input (rainfall + irrigation) of potatoes in northwest China was 498 to 520 mm, and potassium application was 201 to 393 kg·ha^−1^, according to the binary quadratic regression model. A binary quadratic regression model was developed by Li [27] to assess the effects of nitrogen and irrigation on sugar beet yield. The findings suggested that the highest yield required irrigation of 2453.03 m^3^·ha^−1^ and nitrogen application of 123.49 kg·ha^−1^ under fertilizer base and film drip irrigation in inner Mongolia.

Sugar beets require a large amount of water and fertilizer during their growth [27,28]. The fertilizer and water supply models were designed by farmers according to maximum yield when cultivating sugar beet. The models had a detrimental effect on the sustainable development of agriculture due to high costs, consumption, pollution, and other issues [27]. Currently, drip irrigation fertilization technology has been utilized and popularized. However, many contemporary studies focus on the separate effects of water and fertilizer and the interaction between irrigation and single fertilizer. Few studies have been conducted into the interaction effects of water and fertilizer on drip fertilization of sugar beets. In this study, the influence of irrigation and fertilization reduction on yield, quality, water, and fertilizer use efficiency of sugar beet was studied. By developing a binary quadratic regression model (BQRM) to characterize the appropriate water and fertilizer dosage range of each index. A water and fertilizer supply model that can comprehensively optimize the yield and water and fertilizer use efficiency of the study area was proposed using these intervals. This study provides data to support the theoretical basis for high quality, yield, and low cost. It is conducive to the sustainable development of sugar beet in Northern China.

## 2. Materials and Methods

### 2.1. Description of Experimental Plots

**Figure 1:**
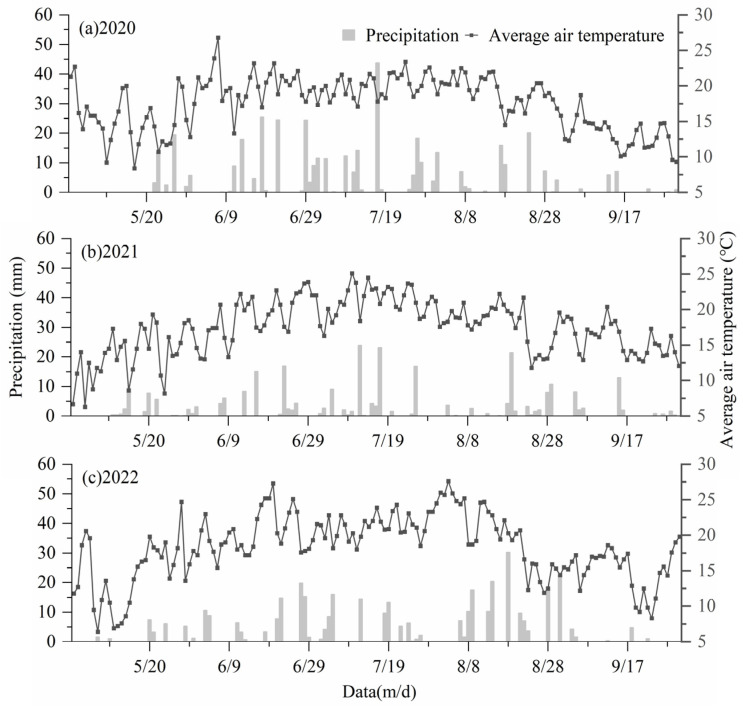
Daily average air temperature and precipitation during the growth period of sugar beet from 2020 to 2022.

This research was conducted between 2020 and 2022 at the Wulanchabu Institute of Agricultural and Forestry Sciences, Wulanchabu City, Inner Mongolia (40.9232° N, 13.1196° E), at an altitude of 1425.3 m. The local arid climate was continental. The annual average precipitation was 384 mm. The average temperature was 4.4 °C. The ≥10 °C accumulated temperature was 2970 °C. The annual sunshine duration was 2980.9 h. The frost-free period was 115 days. The soil was chestnut soil in the 0–80 cm layers. Soil characteristics found in the experimental field are outlined in Table 1. The precipitation and average temperature during the growth period of sugar beet are illustrated in Figure 1.

### 2.2. Experimental Design

The sugar beet variety “IM1162” (the main cultivar in the test area) was examined in the present study. The test fertilizer was composed of Urea (N 46%), ammonium polyphosphate (N 13%, P_2_O_5_ 68%), and potassium sulfate (K_2_O 50%). The field experiment was performed in a split-plot design with three replicates. Four fertilization levels of no reduction (F3), 10% reduction (F2), 20% reduction (F1), 10% increase (F4), and three irrigation levels of no reduction (W3), 15% reduction (W2), and 30% reduction (W1) were used in the experiment. The traditional irrigation and fertilization levels (the fertilizer amount was determined by local practice; the sum of irrigation and precipitation was 92.35% to 109.16% of the water requirement of sugar beet in the test area) were established as a control (F3W3). The fertilization levels were the primary plots. The irrigation levels (subplots) were established under each primary plot. The total irrigation and fertilization of each treatment are outlined in Table 2. Additionally, three no-fertilizer controls were implemented: no irrigation reduction, 15% reduction, and 30% reduction. The area of each plot was 30 m^2^ (5 m × 6 m). The theoretical number of plants was 83,000 plants·ha^−1^ (rows of 60 cm width, plant spacing of 20 cm). Each plot encompassed 10 rows in length. Transplanting was performed on 15 May when the seedling age was 30 days. The first irrigation and topdressing were performed 40 days after transplanting. Other irrigation and topdressing was conducted once every 25 to 30 days after the first irrigation. Three identical irrigations were performed during the growth period. Fertilizer was administered both before transplanting and during irrigation. The nitrogen fertilizer application ratio was 3:4:2:1. Phosphate and potassium fertilizers were applied at a ratio of 5:2:2:1. The drip belt type was plate-attached, with a dripper spacing of 20 cm, dripper flow rate of 2.3 L/h, and a dripper working pressure of 0.2 MPa. Sugar beets were harvested on 8 October.

### 2.3. Measurements and Methods

#### 2.3.1. Plant Sample Collection and Processing

Sugar beet samples were obtained every 20 days after transplanting. Representative areas were chosen in each plot, and five plants with consistent growth were randomly assessed and collected. With the first leaf scar as the dividing line, the root was separated and weighed.

#### 2.3.2. Yield

Yield was measured according to the standard GB/T 10496-2018 Sugar beet [29].

#### 2.3.3. Sugar Content and Sugar Yield

The brix value was assessed using a Refractometer PAL-1 brix meter (Tokyo, Japan).

Sugar content (%) = brix value × 0.8(1)

Sugar yield (t·ha^−1^) = yield (t·ha^−1^) × sugar content (%) (2)

#### 2.3.4. Water Consumption and Total Water Input

Water consumption was evaluated by the water balance method [30,31]:

*ET* = *I* + *Pr* + *U* − *D* − *R* − Δ*W* (3)

where *ET* is the plant water consumption (mm); *I* is the irrigation in sugar beet growth period (mm); *Pr* is the effective precipitation (mm); Δ*W* is the change of 0–80 cm soil water storage (mm); *R* is the surface runoff (mm); *D* is the deep leakage (mm); *U* is groundwater recharge (mm).

The topography of the test region was flat, the precipitation was limited, and the groundwater burial was more than 40 m. Throughout the test, there was very little deep leakage and little surface runoff. When *R*, *D*, and *U* are minor, they can be disregarded. The above formula is simplified as follows:

*ET* = *I* + *Pr* − Δ*W* (4)

The following formula was employed for the total amount of water input:

Total water input = *I* + *Pr* (5)

#### 2.3.5. Water Use Efficiency (WUE)

*WUE* = *Y*/*ET* (6)

where *Y* is the yield of sugar beet (t ha^−1^).

#### 2.3.6. Irrigation Water Use Efficiency (WUEi)

*WUE* = *Y*/*I* (7)

#### 2.3.7. Fertilization Agronomic Efficiency (FAE)

*FUE* = *Y* − *Y0*/*F* (8)

where *Y0* is the yield of non-fertilized control under the same irrigation (t·ha^−1^); *F* is the total amount of fertilization (N, P_2_O_5_, K_2_O) (kg·ha^−1^).

#### 2.3.8. Fertilizer Partial Factor Productivity (PFP)

*PFP* = *Y*/*F* (9)

#### 2.3.9. Sugar Yield Water and Fertilizer Use Efficiency

The yield in Formulas (6)–(9) was replaced with the sugar yield to compute the sugar yield water use efficiency (SWUE), sugar yield irrigation water use efficiency (SWUEi), sugar yield fertilization agronomic efficiency (SFAE), and sugar yield fertilizer partial productivity (SPFP).

#### 2.3.10. Data Statistics

Data were analyzed using Excel 2003. Analysis of variance (LSD), two-way ANOVA, and correlation analysis (Pearson) were performed using SPSS9.0. Figures were made using Excel 2003, Mathematica 9.0, and Origin 9.0.

## 3. Results

### 3.1. Yield, Sugar Yield, and Quality (Root Sugar Content in Harvest Period)

Sugar beet yield was extremely significantly influenced by the fertilization and irrigation reduction in 2020 and 2021 (*p* < 0.01) and significantly affected by the interaction (*p* < 0.05) (Table 3). The main effects of fertilization and irrigation on yield were significant and extremely significant, respectively, in 2022. The yield of F2W3 increased by 0.5 to 8.01% relative to CK, with the highest increase observed in 2021. F2W2 and F3W2 decreased by 1.20 to 3.92% compared to CK, but the difference was not significant (*p* > 0.05). The yield of each F1 fertilization level and W1 irrigation level treatment was lower than CK over all years, with a significant decrease of 5.79 to 16.49% and 6.58 to 15.28% by F1W1 and F4W1, respectively. The yield of F4W2 over all years was also lower than CK, which was significantly reduced by 7.40% and 4.41% in 2020 and 2021, respectively. The yield could not be raised by a 10% fertilizer increase when irrigation was lowered by 15% to 30%. This further impeded the coordination between water and fertilizer. It prevented beets from utilizing water more efficiently. The fertilizer dosage exceeded the optimal level.

Irrigation and fertilization levels significantly affected the sugar yield and sugar content of the root and had an interaction influence on the sugar content (Table 4). The sugar content of F2W3 was reduced by 6.87% compared to CK. Additionally, the sugar content could be increased by other irrigation and fertilization reduction treatments compared to CK, with F1W1 being the highest. The sugar content was increased by F4W1 and F4W2, with F4W1 showing a significant rise of 3.24 to 10.16%.

There was no significant difference between the sugar yield of F2W2, F2W3, F3W2, and CK over 3 years (*p* > 0.05) (Table 4). However, the sugar yield of F2W3 was increased by 0.46 to 2.02% compared to CK over all years. The sugar yield of F1 fertilization level treatments was 1.75% to 3.33% and 4.62% to 6.74% lower than CK, respectively, in 2020 and 2021. The sugar yield of W1 irrigation level treatments was 2.14–3.76%, 2.64–6.74%, and 1.20–5.04% less than CK, respectively, in all years. In 2020, this decrease was significant. Sugar yield was reduced by F4W1 and F4W2 compared to CK, with F4W1 significantly lower than CK in 2020 and 2021. Reductions in fertilization and irrigation exerted comparable impacts on yield and sugar yield. The impact on sugar yield was narrowed compared to yield.

### 3.2. Root Fresh Weight and Sugar Content in Each Sugar Beet Growth Period

**Figure 2:**
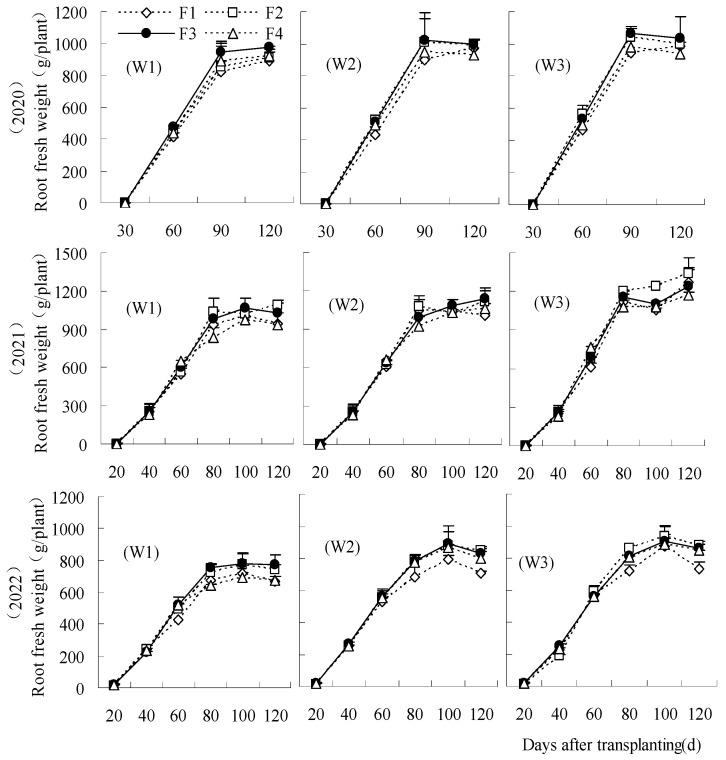
Effects of water and fertilizer reduction on root fresh weight of sugar beet.

Root fresh weight was reduced by the treatments of W1 and W2 irrigation levels compared to CK from 60 to 120 days after transplanting (Figure 2). The reductions of F2W2 and F3W2 were low and had no significant effect on fresh root weight from 90 to 120 days over all years. Similar findings were recorded at low fertilization levels (20% reduction), with F1W1 being the lowest. From 60 days to 120 days after transplanting, the root fresh weight of F1W1 was significantly decreased by 13.98 to 23.01%, 9.21 to 23.55%, and 17.18 to 23.41%, respectively, compared to CK in each year. The root fresh weight could be elevated by F2W3 between 80 days and 120 days after transplanting in 2021 and 2022. The root fresh weight of F2W3 was increased from 20 to 60 days in 2020, but the differences were not significant. F2W3 significantly reduced these levels by 33.33% and 24.02%, respectively, compared to CK at 20 to 40 days in 2022.

**Figure 3:**
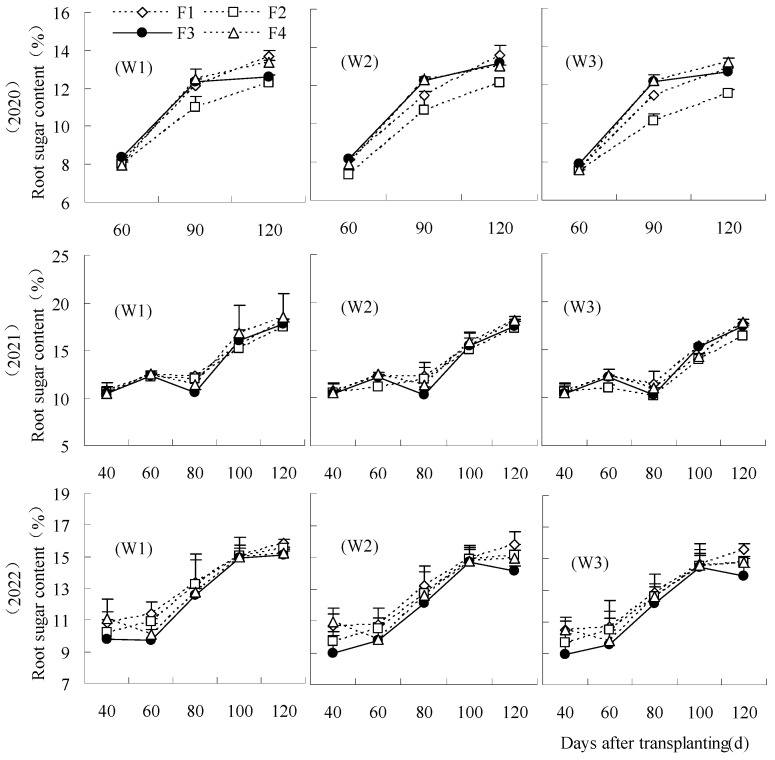
Influence of water and fertilizer reduction on root sugar content.

The sugar content of root was decreased by F2W3 in comparison to CK at 60 days to 120 days in 2020 and 2021 (Figure 3). The decrease was larger in 2020, representing a significant reduction of 4.68% to 16.72%. The sugar content of F2W3 increased by 0.29% to 9.96% compared to CK at each treatment in 2022, but the increase was not significant. The sugar content was not significantly impacted in F3W2 at each period. F4W1 and F4W2 could elevate the root sugar content compared to CK in each period, with F4W1 being the highest.

### 3.3. WUE, WUEi, SWUE, SWUEi

**Figure 4:**
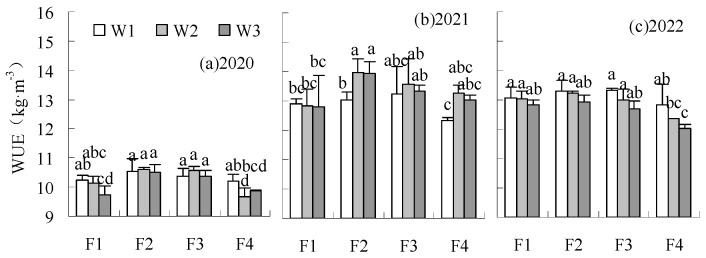
Influence of water and fertilizer reduction on sugar beet WUE. Note: The different lowercase letters represent significant differences between treatments; as below.

**Figure 5:**
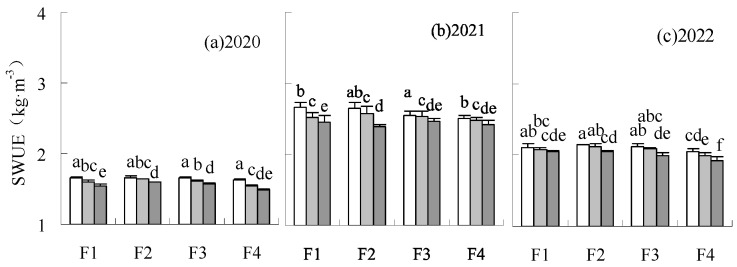
Influence of water and fertilizer reduction on sugar beet SWUE.

The WUE of F2W2, F2W3, and F3W2 was increased by 2.14–4.66%, 1.12–4.43%, and 1.84–2.26%, respectively, compared to CK over all years (Figure 4), but the difference was not significant. The WUE of F2W1 and F3W1 were higher than CK in 2020 and 2022 but lower than CK in 2021. The low irrigation level limited yield formation in a low precipitation climate of 2021. WUE was reduced by treatments under F1 and F4 fertilization levels in 2020 and 2021, with F4W3 significantly reducing it by 2.23% to 5.25% compared to CK. The coordination between fertilization and irrigation was poor when fertilization was lowered by 20% or increased by 10%. These treatments limited the water utilization of sugar beet. The reduction of fertilization and irrigation on SWUE differed from WUE (Figure 5). The SWUE could be increased by the fertilization reduction treatments. The SWUE of the treatments under irrigation reduction treatments were higher than CK when fertilization was decreased, with the SWUE of F1W1 and F2W1 significantly increased by 5.36–8.18% and 4.99–7.89%, respectively. F4W1 was also higher than CK in all years, with a significant increase of 2.91% in 2020. F2W3 had no significant influence on SWUE. The SWUE significantly decreased by 2.55% under F1W3 in 2020 and significantly decreased by 1.61 to 6.17% under F4W3 in all years, compared to CK.

**Figure 6:**
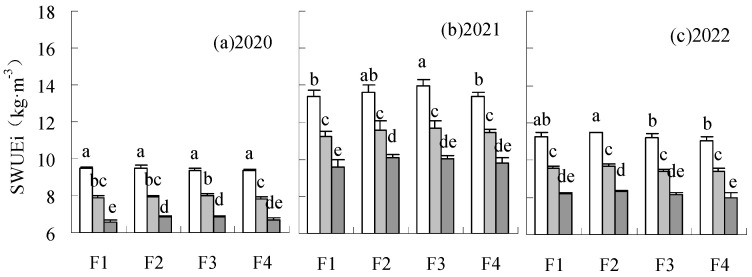
Influence of water and fertilizer reduction on sugar beet WUEi.

**Figure 7:**
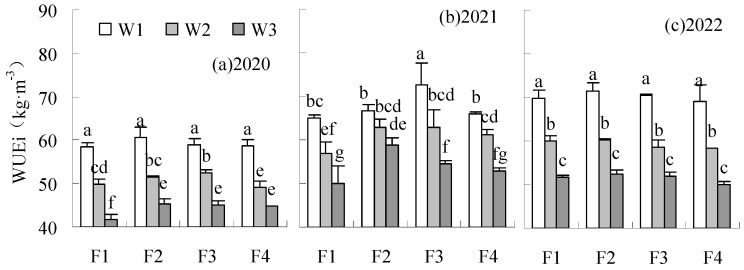
Influence of water and fertilizer reduction on sugar beet sugar yield SWUEi.

WUEi and SWUEi in each treatment were elevated by treatments under W1 and W2 irrigation levels compared to CK (Figure 6 and Figure 7). The treatments under the W1 irrigation level were higher than W2. The treatments under the W1 irrigation level exhibited a significant increase in WUEi of 29.71 to 34.49%, 19.30 to 33.39%, 33.46 to 37.77%, and a significant increase in SWUEi of 29.71 to 34.49%, 19.30 to 33.39%, 33.46 to 37.77%, respectively, relative to CK. WUEi and SWUEi could be elevated by F2W3 compared to CK, with a significant elevation in WUEi of 8.01% in 2021. WUEi and SWUEi were lowered by F1W3 and F4W3. The difference between F1W3 and CK was significant.

### 3.4. FAE, PFP, SFAE, and SPFP

**Figure 8:**
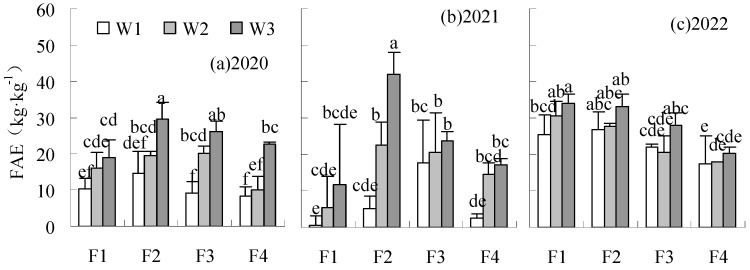
Influence of water and fertilizer reduction on sugar beet FAE.

**Figure 9:**
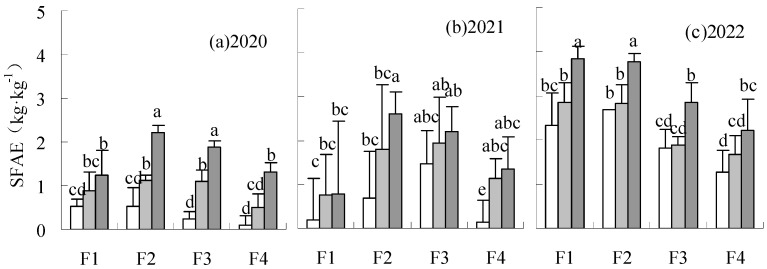
Influence of water and fertilizer reduction on sugar beet SFAE.

The FAE and SFAE were significantly reduced by treatments under W1 and W2 irrigation levels compared to CK in all years (Figure 8 and Figure 9). FAE and SFAE could be increased by F2W3 compared to CK in all years, with a significant increase of 76.85% and 31.57%, respectively, in 2021 and 2022. The SFAE of F1W3 was significantly reduced by 34.35% relative to CK in 2022.

**Figure 10:**
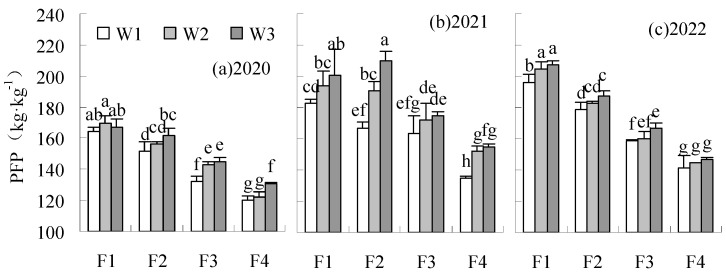
Influence of water and fertilizer reduction on sugar beet PFP.

**Figure 11:**
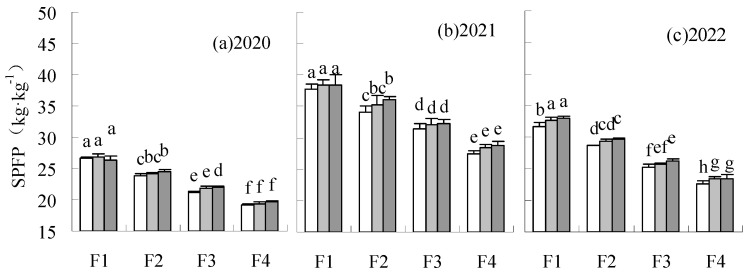
Influence of water and fertilizer reduction on sugar beet SPFP.

PFP and SPFP were significantly elevated by F1 and F2 fertilization levels, compared to CK in 2020 and 2022 (Figure 10 and Figure 11). SPFP was also significantly increased by F1 and F2 fertilization levels treatments in 2021. The PFP of F2W1 reduced by 4.65% relative to CK in 2021, but the difference was not significant. The PFP and SPFP were reduced by F3W1 and F3W2 compared to CK, with a significant decrease of 4.78 to 8.66% by F3W1 in all years. Similar results were found at the F4 fertilization level. The PFP and SPFP of the treatments under the F4 fertilization level were significantly lower than CK.

### 3.5. Interaction Effects of Water and Fertilizer in Drip Irrigation

#### 3.5.1. Relationship Between Yield, Sugar Yield, Irrigation, and Fertilization

A binary quadratic regression model was established based on the least square method. These models were used to explain the relationship between irrigation and fertilization with yield and further analyze the interaction effect of water and fertilizer (Table 5). All binary quadratic regression equations reached significant levels (*p* < 0.05), with coefficients of determination over 0.81. The coefficients of each fitting equation F^2^ and I^2^ were negative. The fitting equation images of yield and sugar yield in all years (the interaction effect of irrigation and fertilizer on yield and sugar yield) exhibited a convex surface, opening downward. The irrigation and fertilization levels of the maximum yield and sugar yield are outlined in Table 5. The fertilization for maximum yield was a reduction of 3.47% and 25.67%, respectively, compared to CK in 2021 and 2022. The irrigation of maximum yield required an increase of 6.19% to 21.98% compared to CK over all years. The fertilization for maximum sugar yield was lower than CK, with a decrease of 2.19% to 12.25%. The irrigation for maximum sugar yield was a reduction of 3.84% in 2020 and an increase of 4.78% and 2.61% in 2021 and 2022, respectively, compared to CK.

#### 3.5.2. Relationship Between Yield, Sugar Yield, Water, and Fertilizer Use Efficiency with Irrigation and Fertilization

**Figure 12:**
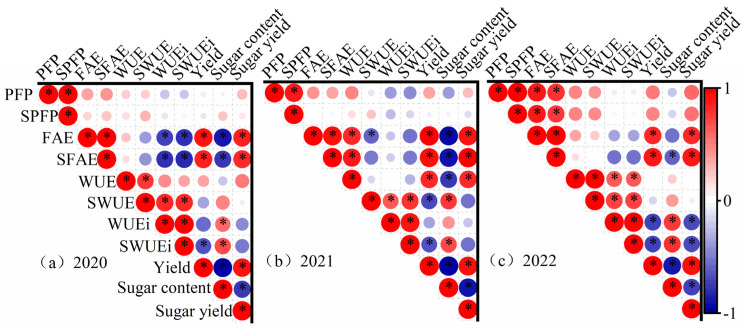
Correlation analysis of yield, quality, and water and fertilizer utilization efficiency. Note: * denotes a significant correlation at *p* < 0.05.

A significant positive relationship was observed between yield and sugar yield (Figure 12). The correlation between yield and sugar content was significantly negative in all years. Similar findings were observed between sugar yield and sugar content. The yield exhibited a significant positive correlation with FAE over all years and a negative correlation with WUEi in 2022. The yield and WUE possess a significant positive correlation in 2021. Water was a limiting factor for high yield in this year. Similar relationships were observed between the sugar yield with water and fertilizer use efficiency, but the significance was reduced. Reductions in irrigation and fertilization had less influence on sugar production than yield. Each year’s relationship between the efficiency of fertilizer and water use varied. FAE and WUEi exhibited a significant negative correlation in 2020. Meanwhile, FAE and WUE exhibited a significant positive correlation in 2021. PFP and FAE, WUE, and WUEi showed significant positive correlations in 2022.

**Figure 13:**
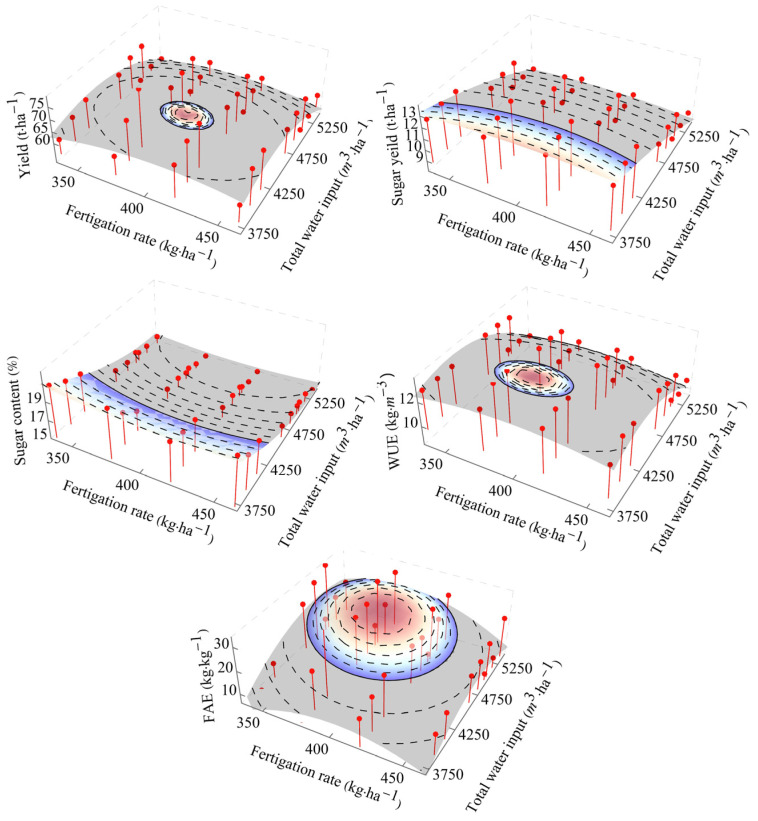
Regression analysis for the response variable of yield, quality, water, and fertilizer use efficiency under total water input and fertigation. Note: the colored portion is the region higher than CK.

The data from each year was compiled using total water input during the growth period. Based on the correlation analysis, FAE and WUE exhibited a stronger correlation with yield or sugar production relative to PFP and WUEi. FAE and WUE have enhanced production-guiding importance. Thus, further analyses were performed on yield, sugar content, sugar yield, FAE, and WUE. As observed, the interaction effects of total water input and fertilizer application on yield, WUE, and FAE provide a downward convex curve. At the same time, the interaction effect on sugar yield and content was a broken surface with increasing or decreasing slopes (Figure 13). The primary effect of fertilization on sugar content and sugar yield was lower than the impact on yield.

**Figure 14:**
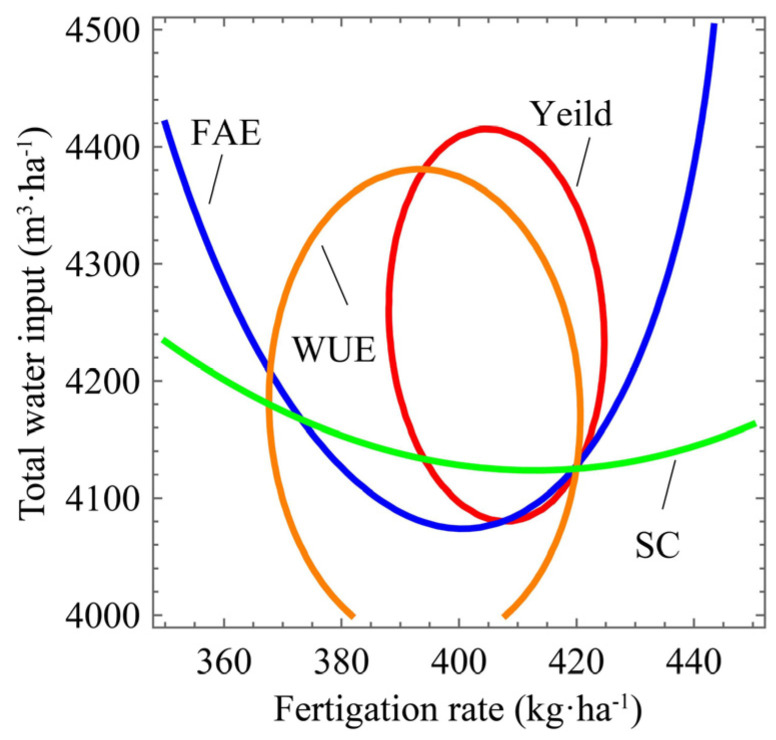
Yield, quality, water, and fertilizer use efficiency comprehensive optimization analysis. Note: SC mean root sugar content over harvest time.

As observed, the maximum value of each index could not be obtained from the same water and fertilizer levels (Figure 13 and Figure 14). The appropriate region of total water input and fertilization in the test area was bordered, with the purpose of not lowering production, quality, and water and fertilizer use efficiency (Figure 14). The region was surrounded by yield, sugar content, and FAE.

The intersection of the yield curve and sugar content curve was the minimum water consumption. This model’s total water input and fertilization were 4136.46 m^3^·ha^−1^ and 408.03 kg·ha^−1^. The intersection of the yield curve and sugar content curve was the minimum fertilization, with the fertilizer amount being 394.04 kg·ha^−1^, and the total water input was 4380.82 m^3^·ha^−1^.

## 4. Discussion

The crop yield, water, and fertilizer use efficiency can be enhanced by drip fertilization [6,32,33]. The yield of sugar beet was not significantly influenced by the appropriate reduction of water and fertilizer under these technical conditions [7,34]. Reducing fertilization by only 10% or irrigation by 15% did not significantly reduce sugar beet yield or sugar production. The yield under 10% fertilization reduction was higher than control. However, the yield did not increase with irrigation reduction. The yield demonstrated a significant decrease under a 20% reduction in fertilization and a 30% reduction in irrigation. Moreover, the sugar yield decreased when subjected to treatments involving a 30% reduction in irrigation. Crops were further constrained by water in a water-constrained ecosystem [35,36,37]. The sugar beet demand for fertilizer was reduced by drip fertilization; however, the water demand was not reduced. Previous studies have determined that the root sugar content of sugar beet gradually increased with decreased irrigation [18,38] and fertilization [39]. In this experiment, the quality of sugar beet could be enhanced through a reduction in irrigation and fertilizer application, with the exception of a 10% decrease in fertilization. The sugar content of the root could be increased by those treatments at 90–120 days following transplanting. Consistent with Elmasry et al.’s [40] findings, a significant inverse correlation between yield and sugar content was identified. The reduction of fertilization and irrigation decreased the fresh weight of the root. Lower levels of sucrose were needed to achieve higher sugar concentrations. The fresh weight of the root was significantly improved by the treatment with 10% fertilization reduction through the accelerated growth of the root during the root tubers and sugar growth stage (60 days to 100 days). Sugar consumption and sucrose accumulation were increased by these factors.

The impact of irrigation on WUE differs from that of previous studies. According to some researchers, crop WUE could be significantly increased by reducing irrigation levels [22,41,42]. Gheysari et al. [43] claimed that WUE and WUEi of silage maize could not be significantly affected by deficits in irrigation. This study demonstrated that WUEi could be significantly increased by irrigation reduction relative to control. WUE was increased by the treatments of 15% reduction in irrigation, but the difference was not significant. The treatments of 30% irrigation reduction reduced WUE in years with lower rainfall (2021). According to previous research, fertilizer use efficiency decreased with the increase in fertilizer application amount [44,45,46,47]. The results of our study were different from those of previous studies. PFP could be significantly elevated by reducing fertilization. FAE could increase with a 10% decrease in fertilization. Conversely, it was significantly reduced by the treatments of 20% reduction in 2020. The fertilizer productivity was reduced by poor fertilization when the rainfall was high. In 2021, the yield and quality were significantly related to WUE because of the low precipitation. The influence of irrigation on sugar beet was more significant than fertilization in the study area. In 2022, WUEi exhibited a significant negative correlation with FAE. Excessive high water input is not conducive to fertilizer use in sugar beet.

As demonstrated previously, yield response to the interaction between irrigation and fertilizer level presented a significant bivariate quadratic regression [14,25,26]. The water use efficiency was elevated by fertilization when the fertilization increased the yield. The functions of irrigation are similar to fertilization. This study demonstrated that the FAE and PFP of sugar beet were lowered by irrigation reduction. WUE and WUEi were decreased by the 20% reduction in fertilization treatments compared to the control. The treatment with a 10% increase in fertilization reduced sugar beet yield, water, and fertilizer use efficiency relative to the control. The results indicated that the coordination between water and fertilizer was more important for the high-yield and efficient cultivation of sugar beet than mutual promotion. High yield and efficient sugar beet cultivation should be guaranteed by a sensible reduction in fertilization when the water input is insufficient.

Regression analysis was employed to characterize the water and fertilizer ranges with the purpose of high yield and efficiency in this study. The irrigation should be elevated to meet the demands of sugar beet when the amount of water and fertilizer is set according to the maximum yield. High irrigation produces a worse water use efficiency. The fresh weight of the root did not vary significantly after 90 to 120 days of transplanting. The adjustment of the third irrigation amount (90 days after transplanting) was conducive to further reducing the total irrigation. The fertilization demand was reduced when the water and fertilizer models were formulated according to the maximum sugar yield. In 2020, the demand for irrigation was also reduced by 3.84%. The sensitivity of sugar production, SFAE, SPFP, and SWUEi to water and fertilizer reduction was lower than the yield. There is more range for water and fertilizer reduction in the model developed according to sugar production. A reasonable range of water and fertilizer was formulated for the purpose of not reducing the yield, water, and fertilizer use efficiency. The ranges of each index were superimposed to identify the optimal irrigation and fertilization mode in the test area. The suitable level of total water input was 4136.46 to 4380.82 m^3^·ha^−1^, and the amount of fertilization was 394.04 to 408.03 kg·ha^−1^. The average rainfall was 309.46 mm during the sugar beet growth period in the experiment. The corresponding appropriate irrigation volume was 1041.91 to 1286.27 m^3^·ha^−1^.

## 5. Conclusions

To reduce the amount of water and fertilizer of sugar beet and improve the use efficiency, this experiment investigated the responses of sugar beet to the reduction of water and fertilizer under drip fertilization from 2020 to 2022. The results indicated that the yield, sugar yield, water, and fertilizer use efficiency were not significantly reduced under a 15% irrigation reduction and 10% fertilization reduction. The yield and sugar yield decreased with a 30% irrigation reduction and a 20% fertilization reduction. The irrigation reduction had a higher decrease of 6.58 to 15.28% and 2.14 to 6.74%, respectively, compared to fertilization reduction. When the irrigation was reduced, the yield decreased by the increased fertilization treatment with a reduction of 4.41 to 15.28% compared to the control. Increasing fertilization could not alleviate the adverse effects of insufficient irrigation on sugar beet. The sugar content of root in harvest time could be increased by irrigation fertilization reduction (except the 10% fertilization reduction) compared to the control, with F1W1 having the highest increase, which was significantly increased by 3.64 to 11.73%. PFP was increased by fertilization reduction relative to control. WUEi and SWUE increased alongside the reduction in irrigation. The FAE of irrigation reduction treatments was lower than the control. WUE and FAE were increased by the 10% fertilization reduction treatment with an increase of 1.12 to 4.43% and 14.27 to 76.85%, respectively. The WUE was reduced by the treatments of the 20% decrease and 10% increase in fertilization relative to the control. The influence of these treatments on SFAE, SPFP, and SWUEi was similar to those of FAE, PFP, and WUEi, but the amplitude was reduced. To ensure high quality, high efficiency, and high yield of sugar beet in the test area, the appropriate irrigation level of sugar beet in Northern China was 1041.91 to 1286.27 m^3^·ha^−1^, and the fertilization level was 394.04 to 408.03 kg·ha^−1^.

## Figures and Tables

**Table 1:** Soil characteristics in the experimental field.

	OM	TON	TOP	TOK	Avail.N	Avail.P	Avail.K	pH	EC μs·cm^−1^
	g·kg^−1^	g·kg^−1^	g·kg^−1^	g·kg^−1^	mg·kg^−1^	mg·kg^−1^	mg·kg^−1^		
2020	32.19	2.09	1.05	20.91	134.11	169.88	257.50	7.60	206.00
2021	32.02	2.07	0.97	22.11	134.91	197.50	194.91	7.98	198.40
2022	33.90	2.64	1.12	17.11	128.77	183.27	223.49	7.92	169.30

**Table 2:** The irrigation and fertilization levels of each treatment.

Treatment	Fertilization (kg·ha^−1^)			Irrigation
	N	P (P_2_O_5_)	K (K_2_O)	(m^3^·ha^−1^)
F1W1	96	120	120	945
F1W2	96	120	120	1147.5
F1W3	96	120	120	1350
F2W1	108	135	135	945
F2W2	108	135	135	1147.5
F2W3	108	135	135	1350
F3W1	120	150	150	945
F3W2	120	150	150	1147.5
F3W3(CK)	120	150	150	1350
F4W1	132	165	165	945
F4W2	132	165	165	1147.5
F4W3	132	165	165	1350

**Table 3:** Sugar beet yield across each treatment (t·ha^−1^).

Year	Irrigation Rate	Fertilization Rate			
		F1	F2	F3	F4
2020	W1	55.21 ± 0.93 c	57.25 ± 2.23 bc	55.55 ± 1.38 c	55.52 ± 1.23 c
	W2	57.11 ± 1.46 bc	59.07 ± 0.41 ab	60.07 ± 0.88 a	56.32 ± 1.68 c
	W3	56.22 ± 1.73 c	61.12 ± 1.70 a	60.81 ± 1.21 a	60.39 ± 0.21 a
	F: ** W: ** F × W: *				
2021	W1	61.36 ± 0.81 f	63.05 ± 1.35 ef	68.60 ± 4.84 bcd	62.25 ± 0.60 f
	W2	65.28 ± 2.93 def	72.01 ± 2.43 bc	72.14 ± 4.57 bc	70.20 ± 1.46 bc
	W3	67.50 ± 5.52 cde	79.36 ± 2.36 a	73.47 ± 1.06 b	71.38 ± 0.88 bc
	F: ** W: ** F × W: *				
2022	W1	65.90 ± 1.85 de	67.46 ± 1.88 bcde	66.61 ± 0.31 cde	65.35 ± 3.57 e
	W2	68.84 ± 1.43 abcd	69.11 ± 0.34 abcd	67.21 ± 2.03 bcde	66.87 ± 0.09 bcde
	W3	69.59 ± 0.87 abc	70.70 ± 1.34 a	69.95 ± 1.48 ab	67.63 ± 0.70 abcde
	F: * W: ** F × W: ns				

Note: F1: 20% fertilization reduction; F2: 10%; F3: no reduction; F4: 10% increase; W1: 30% irrigation reduction; W2: 15%; W3: no reduction; different letters following values indicate a significant difference (*p* < 0.05); F represents fertilization; W represents irrigation; F × W indicates the interaction between fertilization and irrigation; ** denotes a significant difference at *p* < 0.01; * denotes a significant difference at *p* < 0.05; ns indicates the difference is not significant (*p* > 0.05); different lowercase letters represent significant differences between treatments; as below.

**Table 4:** Sugar yield and root sugar content at the harvest period of each treatment.

	Sugar Content (%)			Sugar Yield (t·ha^−1^)		
	2020	2021	2022	2020	2021	2022
F1W1	16.23 ± 0.18 a	20.63 ± 0.31 a	16.28 ± 0.04 a	8.96 ± 0.05 def	12.66 ± 0.32 d	10.63 ± 0.24 ab
F1W2	15.79 ± 0.24 bc	19.73 ± 0.40 ab	15.96 ± 0.10 cd	9.02 ± 0.14 cdef	12.87 ± 0.31 bcd	10.97 ± 0.15 ab
F1W3	15.78 ± 0.16 bc	19.18 ± 1.02 bc	15.92 ± 0.06 d	8.87 ± 0.19 ef	12.91 ± 0.56 bcd	11.08 ± 0.09 ab
F2W1	15.69 ± 0.35 bcd	20.39 ± 0.40 a	16.10 ± 0.11 bc	8.98 ± 0.16 cdef	12.86 ± 0.40 cd	10.86 ± 0.01 ab
F2W2	15.48 ± 0.09 cde	18.47 ± 0.14 c	16.04 ± 0.13 cd	9.15 ± 0.04 abcd	13.30 ± 0.55 abc	11.08 ± 0.16 ab
F2W3	15.20 ± 0.34 ef	17.18 ± 0.43 d	15.92 ± 0.06 d	9.29 ± 0.07 a	13.63 ± 0.19 a	11.21 ± 0.08 a
F3W1	15.99 ± 0.28 ab	19.29 ± 0.86 bc	16.09 ± 0.04 bcd	8.88 ± 0.07 ef	13.21 ± 0.33 abcd	10.60 ± 0.18 ab
F3W2	15.28 ± 0.05 def	18.66 ± 0.71 c	16.06 ± 0.11 bcd	9.18 ± 0.12 abc	13.44 ± 0.43 ab	10.81 ± 0.08 ab
F3W3	15.19 ± 0.25 ef	18.47 ± 0.30 c	15.70 ± 0.08 e	9.24 ± 0.07 ab	13.57 ± 0.24 a	10.99 ± 0.18 ab
F4W1	15.91 ± 0.20 abc	20.34 ± 0.50 a	16.21 ± 0.11 ab	8.83 ± 0.10 f	12.66 ± 0.23 d	10.43 ± 0.23 b
F4W2	15.90 ± 0.22 abc	18.74 ± 0.60 bc	16.13 ± 0.08 abc	8.95 ± 0.15 def	13.15 ± 0.21 abcd	10.78 ± 0.21 ab
F4W3	15.00 ± 0.15 f	18.58 ± 0.26 c	15.99 ± 0.11 cd	9.06 ± 0.10 bcde	13.26 ± 0.35 abc	10.81 ± 0.32 ab
F	**	**	*	**	*	**
W	**	**	**	**	**	**
F × W	*	*	*	*	ns	ns

**Table 5:** Regression analysis for response variables of yield, sugar yield under irrigation, and fertigation in all years.

Year	Quadratic Regression Equation	R^2^	*p* Value	Y (Max)	Y (Max) Point	
					F	I
2020	Y1 = −30.206 + 0.414F − 6.194 × 10^−4^F^2^ + 1.471 × 10^−4^I + 7.633 × 10^−5^FI − 9.298 × 10^−6^I^2^	0.872	*	61.752	420.30	1433.61
2021	Y2 = −248.365 + 1.195F − 1.44 × 10^−3^F^2^ + 0.114I − 1.44 × 10^−5^FI − 3.748 × 10^−5^I^2^	0.857	*	76.537	405.43	1446.28
2022	Y3 = −1.035 + 0.273F − 3.244 × 10^−4^F^2^ + 0.023I − 2.428 × 10^−5^FI − 2.63 × 10^−6^I^2^	0.924	**	71.711	312.20	1646.73
2020	Y4 = 1.695 + 0.032F − 4.857 × 10^−5^F^2^ + 1.510 × 10^−3^I + 5.894 × 10^−6^FI − 1.468 × 10 − 6I^2^	0.800	*	9.208	406.26	1298.06
2021	Y5 = −8.781 + 0.091F − 1.176 × 10^−4^F^2^ + 5.138 × 10^−3^I + 3.816 × 10^−6^FI−2.370 × 10^−6^I^2^	0.965	**	13.625	411.14	1414.63
2022	Y6 = 1.242 + 0.030F − 3.931 × 10^−5^F^2^ + 6.205 × 10^−3^I − 1.045 × 10^−6^FI − 2.101 × 10^−6^I^2^	0.904	**	11.146	368.53	1385.17

Note: Y1, Y2, and Y3 indicate 2020, 2021, and 2022 yields, respectively (t·ha^−1^); Y4, Y5, and Y6 indicate sugar yield (t·ha^−1^); F indicates fertilization (kg·ha^−1^); I denotes irrigation (m^3^·ha^−1^); ** denotes a significant level at *p* < 0.01; * denotes a significant level at *p* < 0.05.

## Data Availability

All data are contained within the article.
